# Broadening the H5N3 Vaccine Immunogenicity against H5N1 Virus by Modification of Neutralizing Epitopes

**DOI:** 10.3390/v10010002

**Published:** 2017-12-23

**Authors:** Subaschandrabose Rajesh Kumar, Sharenya Chelvaretnam, Yunrui Tan, Mookkan Prabakaran

**Affiliations:** Temasek Life Sciences Laboratory, 1 Research Link, National University of Singapore, Singapore 117604, Singapore; cahrajeshkumar@gmail.com (S.R.K.); csharenya@hotmail.com (S.C.); yunrui@smart.mit.edu (Y.T.)

**Keywords:** highly pathogenic avian influenza H5N1 (HPAI-H5N1), neutralizing epitopes, epitope modified vaccine, antibody-guided design

## Abstract

The highly pathogenic avian influenza (HPAI) H5N1 virus remains to be one of the world’s largest pandemic threats due to the emergence of new variants. The rapid evolution of new sub-lineages is currently the greatest challenge in vaccine development. In this study, we developed an epitope modified non-pathogenic H5N3 (A/duck/Singapore/97) vaccine for broad protection against influenza H5 subtype. H5N3 hemagglutinin (HA) mutant reassortant viruses with A/Puerto Rico/8/34 (PR8) backbone were generated by mutating amino acids at the 140th loop and 190th α-helix of hemagglutinin. The cross-neutralizing efficacy of reverse genetics-derived H5N3HA (RG-H5N3HA) mutants was confirmed by testing reactivity with reference chicken anti-H5N1 clade 2 virus sera. Furthermore, RG-H5N3HA mutant immunized mice induced cross-neutralizing antibodies and cross-protection against distinct H5N1 viral infection. Our findings suggest that the use of non-pathogenic H5 viruses antigenically related to HPAI-H5N1 allows for the development of broadly protective vaccines and reduces the need for biosafety level 3 (BSL3) containment facilities.

## 1. Introduction

Highly pathogenic avian influenza (HPAI) H5 subtype viruses cause serious outbreaks among chickens resulting in major economic losses in the poultry industry [1]. Vaccination remains to be the best method to prevent and control influenza. However, rapid emergence of antigenically drifted H5N1 viruses, timeline for vaccine strain selection, poor growth properties of selected vaccine strains and the need for high-level biocontainment facilities in vaccine manufacturing are major drawbacks of current influenza vaccine development [2]. Several research groups have tried to generate broadly protective immunogenicity via immunization with a combination of multiple hemagglutinins (HA) of H5N1 viruses [3,4]. However, producing multivalent vaccines significantly increases vaccine cost and delays vaccine production. Thus, the use of a simple and broadly protective monovalent vaccine strain development method against H5 influenza will greatly reduce both the cost of vaccine development as well as the production time. Several research groups, in an effort to improve cross-protective efficacy of vaccine, have evaluated ancestral and consensus HA sequence-based vaccine designs [5,6]. However, the input sequences do not accurately reflect the genetic diversity of H5N1 influenza virus.

Our present study thus focuses on generating broadly protective monovalent vaccine against H5N1 by modifying major neutralizing epitopes of HA, utilizing neutralizing monoclonal antibody (n-mAb) epitopes previously identified [7]. In this study, we used the HA of a non-pathogenic variant, influenza H5N3 (A/duck/Singapore/3/1997), which is antigenically similar to that of H5N1, as the backbone for the development of a broadly protective vaccine. Previous studies have shown that it induces cross protective immunogenicity against human H5N1 viruses from 1997 to 2004 [8]. However, the antigenic site of this non-pathogenic H5 vaccine strain does not match most H5N1 strains, especially that of clade 2 and its sub-lineages, which are known to predominantly circulate and cause human infections [9].

In this present study, we modified the antigenic site by mutating amino acids in the 140th loop and the 190th α-helix of the HA of H5N3 (A/duck/Singapore/97) vaccine strain, which are located near the receptor binding site [7] and contribute to the strain-specific antigenic profile of the H5 viruses [10]. These modified HA were used to generate H5N3/PR8 reassortant virus which were then evaluated their neutralizing efficacy against H5N1 variants in a mouse model.

## 2. Materials and Methods

### 2.1. Ethics Statement

Experimental protocols were reviewed and approved by the Institutional Animal Care and Use Committee (IACUC) of Temasek Life Sciences Laboratory, National University of Singapore, Singapore (IACUC approval numbers TLL-16-025, TLL-17-019).

### 2.2. Viruses

The reassortant H5N1/PR8 viruses listed in Table 1 were previously generated by reverse genetics (RG) [11]. The listed H5N1 and H5N3 viruses (RG-H5N3, RG-H5N3_M0_, RG-H5N3_M1_ and RG-H5N3_M2_) were inactivated with binary ethylenimine as described previously [12].

### 2.3. Vaccine Construction

In this study, we used monoclonal antibodies (3H11, H and 3E8) recognizing neutralizing epitopes of H5HA for vaccine design. The neutralizing conformational epitopes of HA were previously mapped by the characterization of escape mutants with n-mAbs [7,13]. The n-mAb 3H11 selected escape mutants at amino acid position 139 glycine or 140 serine (140th loop), n-mAb H selected escape mutants at amino acid positions 141 proline (140th loop) or 152 lysine, whereas n-mAb 3E8 selected escape mutants at amino acid position 189 arginine in the 190th α-helix (H5 numbering). We substituted amino acids at positions 138–141 and 189 in the HA of H5N3 (A/duck/Singapore/3/97) with amino acids found within the neutralizing epitopes of most clade 2 H5N1 viruses by site directed mutagenesis (Agilent Technologies, La Jolla, CA, USA). The resulting HA mutant with amino acid 189 (K to R) was designated H5N3HA _M0_, mutant with amino acids NGRS replaced by QGSP at position 138–141 was designated H5N3HA_M1_ and mutant with amino acids NGRS replace by QGSP at position 138–141 and 189 (K to R) was designated H5N3HA_M2_. Then, a reassortant viral vaccine containing HA (EF619972.1) or mutant HA (HA _M0_, HA_M1_ and HA_M2_) and neuraminidase (NA) (AY207510) from H5N3 and the six internal genes from A/Puerto Rico/8/1934 (PR8) viruses were generated [11], and designated RG-H5N3, RG-H5N3HA_M0_, RG-H5N3HA_M1_ and RG-H5N3HA_M2_. The reactivity patterns of the vaccine constructs were subsequently confirmed with n-mAbs by immunofluorescence assay (IFA) [14] and the fluorescence signal was detected with an inverted fluorescence microscope (Olympus, London, UK). Next, we evaluated the replication efficacy of RG-H5N3 and its mutants in MDCK cells and embryonated chicken egg by HA titer [15].

### 2.4. Confirmation of Antigenic Relatedness

Antigenic relatedness of vaccine constructs to clade 2 H5N1 viruses was then tested by viral neutralization study using reference chicken anti H5N1 sera (Table 1). Chicken antisera against various H5N1 subtypes were produced in our previous study [11]. Briefly, groups of White Leghorn chickens (*n* = 5), were injected intramuscularly with inactivated H5N1 subtype (Table 1) viruses emulsified in Montanide ISA-70 (SEPPIC, Paris, France) adjuvant. Chicken serum was collected 10 days after the second immunization. We then used these chicken sera to measure the vaccine constructs (H5N3-HA, H5N3-HA_M0_, H5N3-HA_M1_ and H5N3-HA_M2)_ antigenic relatedness to clade 2 H5N1 virus by microneutralization assay [16].

### 2.5. Mice Immunization and Challenge

Specific-pathogen free female BALB/c mice (6 weeks old) were obtained from the Laboratory Animals Centre, National University of Singapore. Mice (10 per group) were vaccinated subcutaneously at days 0 and 28 with 100 µL (HA titer, 128) of inactivated RG-H5N3, RG-H5N3HA_M1_, RG-H5N3HA_M2_ viruses with Freund’s adjuvant (Sigma-Aldrich, St. Louis, MO, USA). Phosphate-buffered saline (PBS) was administered with the same adjuvant as a reference vaccine control. The mice serum collected 21 days after dose 2 (day 49) was evaluated by hemagglutination inhibition (HAI) titer [17], anti-H5HA (A/Indonesia/5/2005) specific antibody titers by indirect ELISA [13] and VMN [16] against influenza viruses. In a separate study, mice (6 per group) were vaccinated with inactive RG-H5N3 or RG-H5N3HA_M1_ or RG-H5N3HA_M2_ vaccine with Freund’s adjuvant on days 0 and 28. Four weeks after the final immunization, mice were anesthetized intraperitoneal (i.p.) with ketamine (100 mg/kg)/xylazine (20 mg/kg) and intranasally challenged with 50 μL (25 μL per naris) of 5 50% mouse lethal dose (MLD_50_) of A/Hubei/1/2010 RG-H5N1 virus (clade 2.3.2.1). Mice were observed daily to monitor body weight, clinical signs of disease and mortality until day 14 after challenge. Mice were humanely euthanized with CO_2_ inhalation if their body weight dropped to 75% of baseline weights.

### 2.6. Statistical Analysis

The HAI and VMN data are expressed as geometric mean ± standard errors (SE). An unpaired two-tailed Student’s *t*-test was performed to determine the difference in the level of significance between the means of two groups (* *p* = 0.05; ** *p* = 0.01; *** *p* = 0.001).

## 3. Results and Discussion

**Figure 1:**
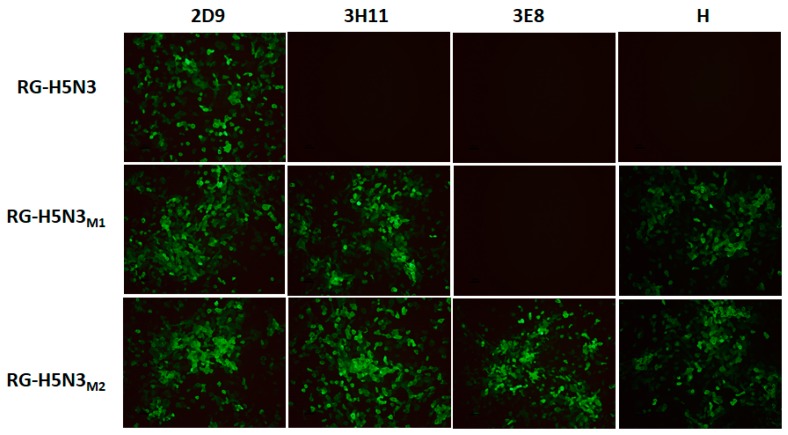
The reactivity of RG-H5N3HA and RGH5N3 HA mutants with H5N1 specific monoclonal antibodies (mAbs). Indirect immunofluorescence assay (IFA) of MDCK cells infected with RG-H5N3 or RG-H5N3HAM1 or RG-H5N3HAM2. Infected cells were fixed and stained with mAb 2D9 (H5 specific positive control) or 3H11 or 3E8, or H.

To broaden the cross protective efficacy of the non-pathogenic H5N3 vaccine candidate, we modified amino acids of major neutralizing epitopes in the 140th loop and 190th α-helix region of HA which have previously been noted as very important determinants of protective immunity to clade-specific influenza H5N1 [7]. Beato et al. reported that the amino acid variations at the loop 140th loop and 190th α-helix may be responsible for antigenic differences of clade 2.2.1, 2.2.1.1 and 2.3.4 of clade 2 H5N1viruses [18]. Also, amino acid substitutions at the solvent-exposed positions 140, 141, and 189 of H5 HA correlate with changes in the antigenic signature of the virus [19]. The 190th α-helix, 220th loop (amino acid residues 221–228) and the 130th loop (residues 134–138) are primarily involved in the formation of receptor binding site. In addition, amino acids in the 220th loop, at positions 226 and 228, have been linked closely to receptor binding specificity of avian or human [20]. Viruses with amino acids Q226 and G228 are specific for the avian (α-2,3 glycosidic linkage) sialic acid receptors, whereas viruses with amino acids L226 and S228 preferentially bind to human receptors (α-2,6 glycosidic linkage). The reactivity patterns of RG-H5N3 or RG-H5N3HA_M1_ or RG-H5N3HA_M2_ were confirmed with H5N1 n-mAbs (3H11, H, 3E8) by IFA (Figure 1). The results showed that RG-H5N3HA_M1_ and RG-H5N3HA_M2_ reacted with mAbs 3H11 and H which recognize conformational epitopes (139 glycine, 141 proline), whereas only RG-H5N3HA_M2_ reacted with n-mAb 3E8 which recognizes conformational epitope (189 arginine) of HA0. These results indicate that the epitope modified H5N3-HA_M1_ and H5N3-HA_M2_ derived from H5N3HA have broader reactivity against previously unreactive H5N1 n-mAbs (3H11, 3E8, and H) produced against clade 2 specific viruses.

We then used the reassortant viruses to measure mutant vaccine efficacy by virus microneutralization assay against reference chicken anti H5N1 sera. Table 1 shows that most of the clade 2 immunized chicken sera neutralization titers were significantly higher and also inhibited epitope modified RG-H5N3HA_M2_ vaccine strain to a larger extent as compared to other mutants (RG-H5N3HA_M0_, RG-H5N3HA _M1_) or RG-H5N3.

This indicates that RG-H5N3_M2_ viruses are more antigenically related to clade 2 H5N1 tested viruses and can thus induce a more significant immune response against clade 2 H5N1 influenza viruses. The reactivity of RG-H5N3_M2_ with reference chicken sera and neutralizing n-mAbs against H5N1 also shows the different antigenic properties from RG-H5N3. Additionally, we tested replication efficacy of RG-H5N3 or RG-H5N3 mutants in MDCK cells. The viral titers of RG-H5N3_M1_ and RG-H5N3_M2_ mutants showed 10^8.3^ TCID_50_/mL and 10^8.0^ TCID_50_/mL on 48 h post infection, respectively. Also, both RG-H5N3 mutant viruses were grown well in embryonated chicken eggs and showed 512 HA titer.

**Figure 2:**
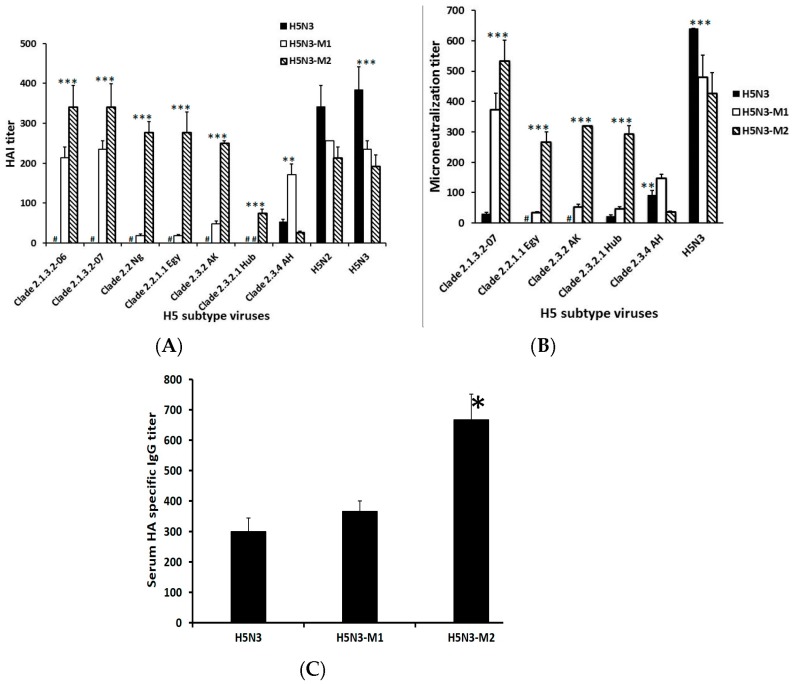
Measurement of systemic immune responses of vaccinated mice sera: Mice subcutaneously immunized on days 0 and 28 with RG-H5N3 or RG-H5N3HA_M1_ or RG-H5N3HA_M2_ viral vaccine. (**A**) Serum hemagglutination inhibition (HAI) assay. The serum HAI on day 49 against H5N1 viruses from clade 2.1 (A/Indonesia/CDC669/2006, A/Indonesia/CDC1031/2007), clade 2.2 (A/Nigeria/6e/2007, A/chicken/Egypt/12186F/2012) and clade 2.3 (A/Whooper Swan/Akita/1/2008, A/Hubei/1/2010, A/Anhui/01/2005). Each point represents the geometric mean titer (*n* = 6) ± standard error (SE). (** *p* < 0.01; *** *p* < 0.001, when compared with RG-H5N3), # represents <8 HAI titer; (**B**) serum virus microneutralization (VMN) assay on day 49 against H5N1 strains. Each point represents the geometric mean titer (*n* = 6) ± standard error SE. (** *p* < 0.01; *** *p* < 0.001, when compared with RG-H5N3), # represents <10 VMN titer; (**C**) measurement of Serum hemagglutinin (HA)-specific immunoglobulin G (IgG) antibody titer on day 49 against H5N1HA protein (A/Indonesia/5/2005) by indirect ELISA. Each point represents the geometric mean titer (*n* = 6) ± standard error SE. (* *p* < 0.05 when compared with RG-H5N3).

Furthermore, immunogenicity and cross-neutralizing efficacy of the epitope modified vaccine was evaluated in a mouse model. The results showed that mice immunized with RG-H5N3HA_M2_ vaccine strain showed significantly (*p* > 0.001) enhanced HAI and cross-neutralizing antibody titers against most H5N1 clade 2 (2.1.3, 2.2.1, 2.3.2) viruses as compared to mice immunized with either RG-H5N3_M1_ or RG-H5N3 vaccine (Figure 2A,B). In addition, mice vaccinated with RG-H5N3_M2_ construct significantly (** *p* < 0.01) induced HA of H5N1 specific immunoglobulin G (IgG) antibody titers when compared with RG-H5N3 vaccinated mice (Figure 2C). Higher antibody titer against H5N1HA could be the result of amino acid mutation in the H5N3HA epitope region at position 189R. The mutated H5N3HA epitope region is homologous to clade 2 H5N1HA, which enhances the strong IgG responses (2-fold higher) when compared to non-homologous epitope region in the RG-H5N3HA.

The amino acid positions 140, 141 and 189 which are found on the surface-exposed membrane-distal receptor-binding region of the globular HA1 are likely to influence HAI titer as they are known to be predominant sites for antigenic variability among clade 2 viruses [19]. Hence, these higher cross-neutralizing antibody titers observed could be the result of the mutated amino acids at 138Q or 140S or 141P and 189R, present in all of the above tested viruses. Several previous studies reported that HAI and cross reactivity of antisera upon vaccination is substantially affected by amino acids located near the receptor binding sites [20,21] and amino acids responsible for antigenic changes [22]. In this study, we constructed the RG-H5N3_M2_ vaccine by mutating three amino acids at positions 140, 141 and 189. These three positions are found on the surface-exposed membrane-distal receptor binding region of the globular HA1, which are also predominant sites for antigenic variability among the clade 2 H5N1 viruses [19] and capable of enhancing antigenic relatedness to clade 2 H5N1 viruses (Table 1). These characteristics may enhance HAI and NT titer of RG-H5N3_M2_ vaccine immunized serum against clade 2 H5N1 tested viruses. However, mice immunized with RG-H5N3_M1_ showed significantly (*p* > 0.01; *p* > 0.05) higher HAI and neutralizing antibody titers against only clade 2.3.4 compared to mice immunized with RG-H5N3_M2_ vaccine, likely due to the variant amino acid at position 189 (lysine). The amino acid arginine or lysine at position 189 on influenza H5 virus hemagglutinin, located in the receptor binding site is one of the major antigenic determinants. Stevens et al. [23] reported that the presence of an arginine at position 189 is characteristic of isolates from clades 2.1 and 2.2 but uncommon in those from clade 2.3.4, which predominantly have a lysine at this position.

Overall, the HAI and neutralizing titer results show that the amino acids at positions 138,140, 141 and 189 are key determinants for inducing cross-neutralizing antibodies against H5N1 viruses.

**Figure 3:**
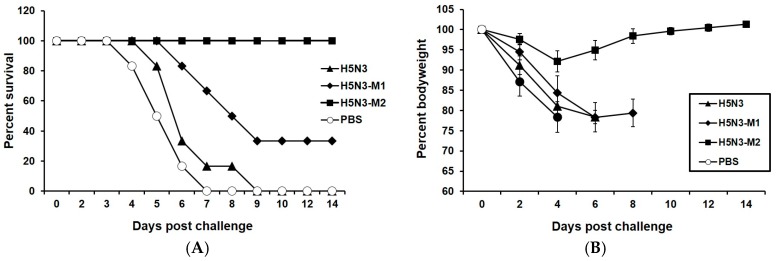
Protection of mice from lethal H5N1 virus challenge. Mice (*n* = 6/group) were subcutaneously immunized on days 0 and 28 with RG-H5N3 or RG-H5N3HA_M1_ or RG-H5N3HA_M2_ viral vaccine and PBS. Four weeks after the second vaccination, mice were intranasally infected with 5 50% mouse lethal dose (MLD_50_) of a clade 2.3.2.1 H5N1 (RG-A/Hubei/1/2010) strain on day 49. Mice were monitored for survival for 14 days and the results were expressed in percent survival (**A**). Weight loss of the mice groups was also monitored throughout the 14-day observation period and the results were expressed in percent body weight compared to the start of the viral challenge (**B**).

In addition, we tested the cross-protective efficacy of epitope modified vaccine approach in a mouse model. Four weeks after the final immunization, all vaccinated mice groups were challenged with 5 MLD_50_ of A/Hubei/1/2010 H5N1 virus (clade 2.3.2.1). Mice immunized with RG-H5N3_M2_ obtained 100% protection (Figure 3A) while RG-H5N3_M1_ vaccinated mice showed only 30% protection. The infection was indicated by rapid loss in body weight of mice immunized with RG-H5N3_M1_ or RG-H5N3 vaccine. However, mice immunized with RG-H5N3_M2_ vaccine showed only mild decrease in body weight (up to 8%) on day 4 and regained their body weight rapidly (Figure 3B). The complete protection of RG-H5N3_M2_ vaccine conclusively showed that the amino acid mutation at the 140th loop (140S and 141P) and 190th (189R) helix in the key epitope region of H5N3HA switches it to a clade 2 specific H5HA antigenic region and enhances the neutralizing antibodies, HAI titer and IgG response against clade 2 specific H5N1 viruses.

Our findings show that non-pathogenic avian influenza viruses can be modified to produce broadly protecting vaccines against circulating HPAI H5 viruses by substituting amino acids based on the variation in the neutralizing epitopes of HA. Moreover, the use of a non-pathogenic-based vaccine as a backbone allows for higher growth titers to be obtained and also eliminates the need to use BSL3 containment facilities for vaccine production.

## Figures and Tables

**Table 1:** Evaluation of antigenic relatedness of reverse genetics-derived H5N3 (RG-H5N3) mutants to H5N1 viruses by virus microneutralization (VMN) assay against chicken anti H5 sera.

Reference Chicken Sera against RG-H5N1 Viruses	Clade	Viral Microneutralization Titer ^#^ 100 TCID_50_ of Virus			
		RG-H5N3	RG-H5N3HA_M0_	RG-H5N3HA_M1_	RG-H5N3HA_M2_
A/Hong Kong/213/03 (H5N1)	1	<10	NT	<10	53.3
A/Vietnam/1203/04	1	16.6	<10	40	<10
A/duck/Thailand/CV-328/2007	1	<10	<10	33.3	<10
A/Indonesia/CDC669/2006	2.1.3.2	<10	30.3	320	403.2
A/Indonesia/CDC1031/2007	2.1.3.2	<10	34.8	253.9	507.9
A/Nigeria/6e/2007	2.2	<10	52.8	100.8	320
A/chicken/Egypt/12186F/2012	2.2.2.1	<10	69.6	25.2	253.9
A/Whooper Swan/Akita/1/2008	2.3.2.1	<10	60.6	50.4	253.9
A/Hubei/1/2010	2.3.2.1	<10	34.8	40	201.6
A/Anhui/01/2005	2.3.4	50.4	<10	126.9	25.2
A/goose/Guiyang/337/2006	4	<10	<10	26.6	<10
H5N3 A/duck/Singapore /3/1997	-	640	367.6	403.2	320
H5N2 A/Common Iora/Indonesia/F89/11/95	-	507.9	NT	403.2	253.9

**^#^** Geometric mean titer (*n* = 3); TCID_50_: tissue culture infectious dose; NT: not tested.
